# Clinical Experience Using a Real Time Autofluorescence Endoscopy System in the Gastrointestinal Tract

**DOI:** 10.1155/DTE.5.119

**Published:** 1999

**Authors:** Tatsuo Ogihara, Haruo Watanabe, Akihiro Namihisa, Osamu Kobayashi, Hiroto Miwa, Nobuhiro Sato

**Affiliations:** Department of Gastroenterology Juntendo University School of Medicine 2-1-1 Hongo Bunkyo-ku Tokyo 113-8421 Japan

## Abstract

Autofluorescence spectra of neoplastic tissues have been reported to be significantly different from those of normal tissues when excited by blue or violet light. From this concept, a light-induced autofluorescence endoscopic imaging system for gastrointestinal mucosa (LIFE-GI; Xillix, Canada and Olympus, Japan) has been newly developed and the clinical evaluation of the prototype system has been conducted in hospitals in Canada, Netherlands and Japan.

We examined the clinical usefulness of the prototype LIFE-GI system for the detection of gastrointestinal cancer and high and low grade dysplasia. The LIFE-GI system was also applied to the early detection of remnant lesions after endoscopic treatment of early gastric cancer and to the detection of laterally spreading superficial colonic tumors.

This system has potential application for the diagnosis of dysplastic lesions and early cancers in the gastrointestinal tract as an adjunct to ordinary white light endoscopy. This system, which needs no administration of a photosensitive agent, may be suitable as a screening method for the early detection of neoplastic tissues.


*Diagnostic and Therapeutic Endoscopy*, Vol. 5, pp. 119–124
Reprints available directly from the publisher
Photocopying permitted by license only

© 1999 OPA (Overseas Publishers Association) N.V.
Published by license under
the Harwood Academic Publishers imprint,
part of The Gordon and Breach Publishing Group.
Printed in Singapore.

### Clinical Experience Using a Real Time Autofluorescence Endoscopy System in the Gastrointestinal Tract

TATSUO OGIHARA, HARUO WATANABE, AKIHIRO NAMIHISA, OSAMU KOBAYASHI, HIROTO MIWA and NOBUHIRO SATO*

*Department of Gastroenterology, Juntendo University School of Medicine, 2-1-1 Hongo, Bunkyo-ku, Tokyo, 113-8421, Japan*

**Autofluorescence spectra of neoplastic tissues have been reported to be significantly different from those of normal tissues when excited by blue or violet light. From this concept, a light-induced autofluorescence endoscopic imaging system for gastrointestinal mucosa (LIFE-GI; Xillix, Canada and Olympus, Japan) has been newly developed and the clinical evaluation of the prototype system has been conducted in hospitals in Canada, Netherlands and Japan.**

**We examined the clinical usefulness of the prototype LIFE-GI system for the detection of gastrointestinal cancer and high and low grade dysplasia. The LIFE-GI system was also applied to the early detection of remnant lesions after endoscopic treatment of early gastric cancer and to the detection of laterally spreading superficial colonic tumors.**

**This system has potential application for the diagnosis of dysplastic lesions and early cancers in the gastrointestinal tract as an adjunct to ordinary white light endoscopy. This system, which needs no administration of a photosensitive agent, may be suitable as a screening method for the early detection of neoplastic tissues.**

*Keywords:* Autofluorescence, Cancer, Dysplasia, Endoscopy, Gastrointestinal tract, Neoplasm

### INTRODUCTION

Neoplastic tissues have different autofluorescence spectra from normal tissues when excited by blue or violet light. This concept has been applied to form an image of neoplastic tissue, such as in early lung cancers [1] and colonic adenomas [2]. Sensitive photodetectors and the increasing capability of microcomputers have improved sensitivity, analytical selectivity, excitation–emission efficiency and ability to apply the properties of autofluorescence spectra to biological systems. Laser induced autofluorescence endoscopic imaging system (LIFE-Lung) was developed for the detection of early cancerous or pre-cancerous lesions in the lung by Xillix Technologies Co. (Canada) and Olympus Optical Co. (Japan) and is used clinically with bronchoscopic procedures.

There are many reports that in the gastrointestinal tract the autofluorescence spectra of dysplasia

* Corresponding author. Tel.: 03 3813 3111, ext. 3305. Fax: 03 3813 8862. E-mail: ogihara@med.juntendo.ac.jp.

and carcinomas are significantly different from those of normal mucosa [3–10]. Based on the LIFE-Lung system, an autofluorescence imaging system for the gastrointestinal (GI) tract (LIFE-GI) has been newly developed by Xillix and Olympus based on the need for a more sensitive and specific method of real time detecting early cancer or precancerous lesion in the GI tract. Initial feasibility studies using a phase-1 prototype LIFE-GI instrument have been performed cooperatively in hospitals in Canada. Netherlands and Japan [11–14]. The usefulness of this system in improving the early detection of GI neoplasm has been demonstrated. For example, esophageal dysplasia invisible under white light endoscopy was detected by fluorescence imaging; dysplastic polyps in the colon appear reddish (abnormal), while hyperplastic polyps appear blue or white (normal).

We applied clinically a prototype LIFE-GI system to gastrointestinal tract and examined the usefulness of this system for the detection of gastrointestinal cancer and high and low grade dysplasia.

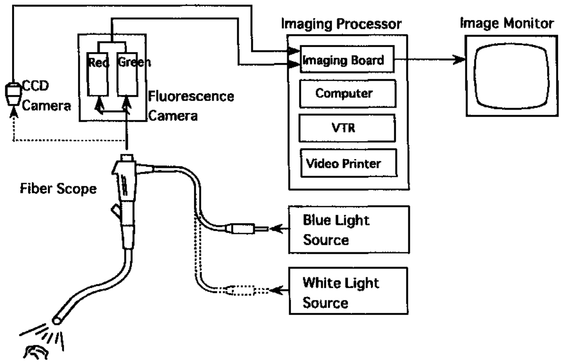

FIGURE 1 Schematic diagram of the real time light-induced autofluorescence endoscopy system.

### MATERIALS AND METHODS

### Autofluorescence Endoscopy System

The LIFE-GI system consists of a filtered blue light source (400–450 nm) for excitation, a white light source for conventional observation, two intensified charge-coupled device (CCD) cameras for the detection of weak fluorescence image, a color CCD camera for conventional white light image, a fiber optical endoscope, a computer based control center, and a monitor [15]. The schematic diagram of the LIFE-GI system is shown in Fig. 1.

The blue excitation light is focused onto the light guide channel of the fiberscope and conducted into the GI tract to illuminate the tissue. Autofluorescence light with longer wavelengths than the excitation light and some backscattered excitation light are collected by the imaging fiber bundle of the endoscope and transmitted to the fluorescence camera which has a beam splitter, filters, and lenses to block the excitation light and to separate the fluorescence light into two wavelength bands: green (490–560 nm) and red (630 nm and longer). The two wavelength bands of light are focused onto two separate intensified CCD cameras: one captures the green image and the other the red image. The video output signals of the two cameras are digitized by the control center and pseudo color image of neoplastic tissue is made and displayed in real time on a color monitor.

### Endoscopic Procedure

For patients scheduled for routine or minute examination endoscopies, systemic endoscopic inspection was carried out first with conventional white light endoscopy (WLE), followed by autofluorescence endoscopic observation (LIFE). The entire procedure was videotaped. After obtaining the LIFE image, biopsy samples were taken from the abnormal areas, identified by white light or fluorescence imaging, as well as from adjacent areas appearing normal on WLE or LIFE.

### RESULTS

### Autofluorescence Images of the Gastrointestinal Lesions

We applied the prototype LIFE-GI system to the lesions in stomach and colon to investigate the clinical usefulness of the system for the detection of neoplastic lesions. In the stomach, the relationship between the color image obtained by LIFE-GI system and the pathology of the biopsied specimen revealed that (i) all of 20 adenocarcinoma appeared dark red in the LIFE image; (ii) three (60%) of five adenomas appeared light red and two adenomas appeared dark red; (iii) 30 (86%) of 35 ulcers and ulcer scars appeared blue in color similar to normal mucosa, while the others appeared light or dark red.

In colonic lesions, the autofluorescence intensity was higher than in lesions of the upper GI tract. Representative LIFE images are demonstrated in Figs. 2–5. Figure 2 shows a case of colonic cancer. The LIFE images of the lesion showed reddish color against the blue color of the normal mucosa. Figure 3 shows a colonic adenoma with a dark reddish color on the LIFE image. Figure 4 shows a hyperplastic polyp with a light reddish color on the LIFE image. Figure 5 is not neoplastic tissue but an inflammatory polyp with whitish blue color similar to normal mucosa in the LIFE image.

The relationship between the color of the autofluorescence image and the pathology of the biopsied specimen in colonic lesions is shown in Table I. Summarized results are: (i) adenocarcinoma appeared dark red; (ii) 84% of adenomas appeared dark red while the remaining appeared

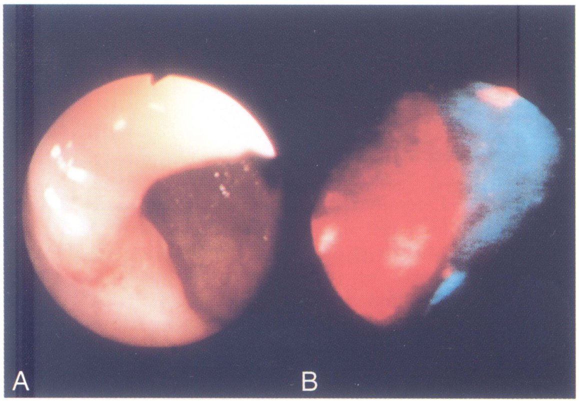

FIGURE 2 Representative endoscopic picture of a colonic cancer. A, conventional white light endoscopy (WLE) image. B, LIFE image. Cancerous lesion showed a reddish color. The bright red color indicated the influence by the fluorescence of the bacteria in the feces on the lesion.

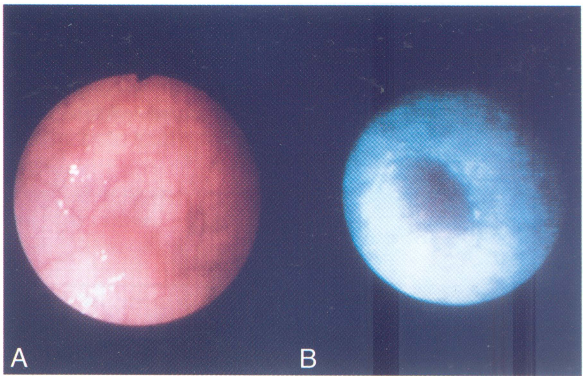

FIGURE 3 Representative endoscopic picture of a colonic adenoma. A, WLE image. The lesion was 6 mm in size and was tubular adenoma with high grade atypia pathologically. B, LIFE image. The lesion showed a dark reddish color.

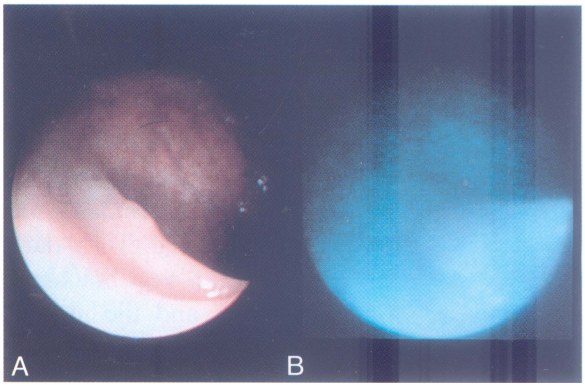

FIGURE 4 Representative endoscopic picture of a hyperplastic polyp. A, WLE image. The lesion was 3 mm in size and was not adenoma but hyperplastic tissue pathologically. B, LIFE image. The lesion showed a light reddish color.

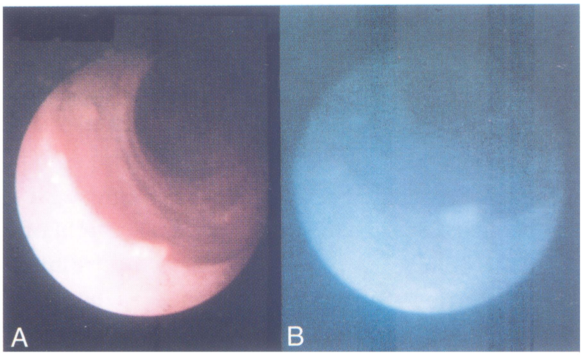

FIGURE 5 Representative endoscopic picture of a pseudopolyp observed in the remission stage of ulcerative colitis mucosa. A, WLE image. The lesion was 3 mm in size and was not neoplastic tissue but inflammatory tissue pathologically. B, LIFE image. The lesion showed whitish blue with color similar to normal mucosa.

TABLE I Relationship between the color image obtained by the LIFE-GI system and the pathological finding of the biopsied specimen

| Pathology (*n*) | Dark red | Light red | Whitish blue |
|---|---|---|---|
| Adenocarcinoma (9) | 9 | 0 | 0 |
| Adenoma (45) | 38 | 7 | 0 |
| Hyperplastic polyp (13) | 0 | 11 | 2 |
| Inflammatory polyp (8) | 0 | 0 | 8 |
| Normal (16) | 0 | 0 | 16 |

light red; (iii) 85% of hyperplastic polyps appeared light red or pink; and (iv) the inflammatory polyps and normal mucosa appeared white or blue.

### Case Presentation

As an example of the clinical application of the LIFE-GI system for the detection of early stage neoplastic lesion, the case of laterally spreading superficial type colonic tumor, which is hard to detect by conventional endoscopy, is demonstrated in Fig. 6A. On the LIFE image (Fig. 6B), a dark reddish area was recognized and a clear boundary was shown between the lesion and the normal mucosa. Biopsied specimen from the dark reddish area showed pathological adenocarcinoma and the lesion was resected surgically. The lesion was 38 × 27 mm in size and was very flat as shown in the loupe photomicrograph (Fig. 6C). Pathological findings of this lesion (Fig. 6D) revealed well differentiated adenocarcinoma (inf. m, v0, ly0).

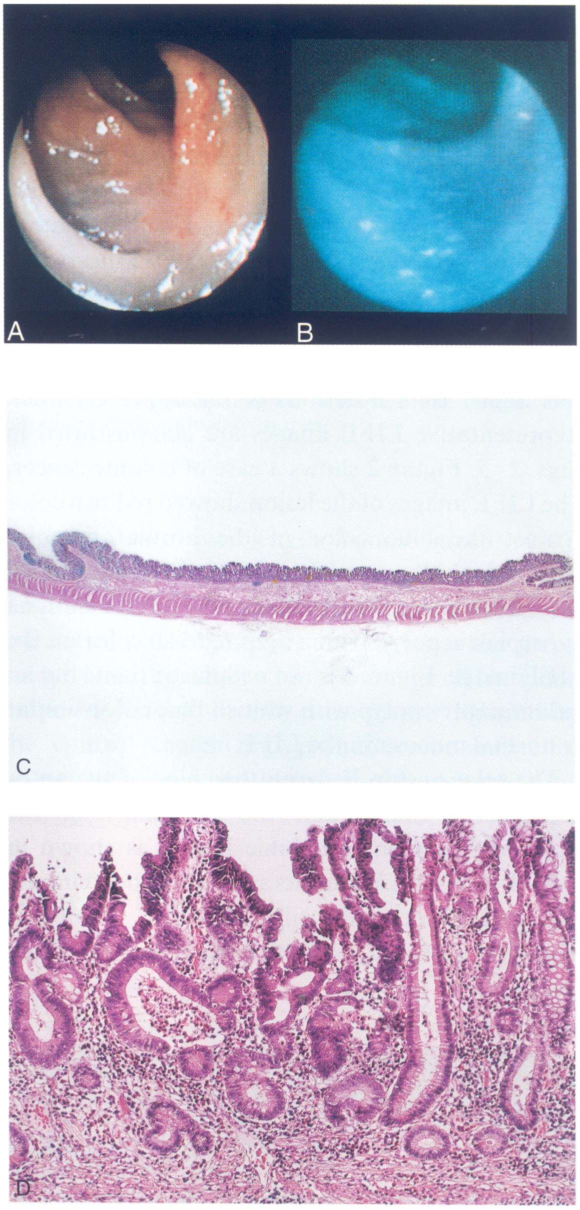

FIGURE 6 Laterally spreading superficial type colonic tumor. A, WLE image; B, LIFE image; C, histologic finding of the resected tumor (× 20) stained with hematoxylin-eosin (the flat lesion can be seen in this cross-sectional view); D, high-power photomicrograph of the mucosal layer. (Pathological finding revealed well differentiated adenocarcinoma.)

### DISCUSSION

Neoplastic tissues, when excited by a light with a specific wavelength, emit autofluorescence light of a longer wavelength. Autofluorescence spectra measured in human normal gastric mucosa had two peaks of both green and red, where the intensity of the green was twice or more that of the red [16]. As compared with normal tissue, cancerous tissue in human stomach and colon showed decreased intensity of green compared to red, suggesting that the autofluorescence of the cancerous tissue seen as dark red was due to the increased intensity of red fluorescence and the decreased intensity of green fluorescence.

Light-induced real time autofluorescence endoscopic imaging (LIFE) is a new technique that has been shown to be of use in discriminating neoplastic from normal tissues. The LIFE-Lung imaging system was already developed for the detection of early cancerous or pre-cancerous lesions in the lung. Recently, clear and reproducible autofluorescence imaging of esophageal, gastric and colorectal neoplasms can be obtained by autofluorescence endoscopic imaging system (LIFE-GI). The clinical usefulness of this system for the early detection of GI tract neoplasms is currently under evaluation. The LIFE -GI system is similar to the LIFE-Lung system with changes to accommodate the characteristics of the GI tract which has wide lumen. The LIFE-GI system has a brighter blue light source, a more sensitive camera and provisions to adjust the relative gain between the red and the green channels (color ratio).

In our examination of the LIFE-GI system, we found it posible to detect gastrointestinal cancer or dysplasia with this system as well as with conventional endoscopy. In the stomach, autofluorescence was observed by the LIFE-GI system in 36 patients with or without gastric cancer and the findings were compared with the findings obtained by conventional endoscopy [17]. Twenty-six lesions, including all 20 carcinomas, showed abnormal fluorescence images as a reddish color on the LIFE-GI monitor and six of the lesions were benign tissues as determined by histological examination.

The system was applied for the detection of the remnant or recurrent lesions of patients after endoscopic treatment of early gastric cancer [14]. Although the diagnosis of complete resection is crucially important in endoscopic treatment for gastric neoplasm, it is sometimes very difficult to determine by conventional endoscopic observation whether the lesion is completely resected. Accordingly, we verified the usefulness of LIFE-GI system in the detection of small remnants of gastric neoplasm after endoscopic resection. Remnants of tumor after resection were suspected in 4 of the 12 lesions by the LIFE-GI system, 2 of which were proven histologically as adenocarcinoma, but could not be detected by conventional endoscopy. The results suggested that small neoplastic lesions, which were difficult to diagnose by conventional endoscopy, could be detected by the LIFE-GI system, which may be useful in the detection of small malignant lesions such as the remnants of endoscopic resected tumors.

TABLE II Indications of the LIFE-GI system on the early diagnosis of gastrointestinal diseases

| |
|---|
| *Esophagus* |
| Superficial type of early esophageal cancer |
| Precancerous lesion of Barrett esophagus |
| *Stomach* |
| Remnant or recurrence after endoscopic treatment of early cancer |
| High risk cancer group in chronic atrophic gastritis |
| *Colon* |
| Superficial type of early colonic tumor |
| Malignant transformation of inflammatory bowel disease |

We also applied the LIFE-GI system to the patients with laterally spreading superficial colonic tumors, which were sometimes difficult to be detected by conventional endoscopy, and the lesions could be accurately diagnosed by the LIFE-GI system [18].

From these clinical experiences, the indications of LIFE-GI system on the early diagnosis of gastrointestinal disease are summarized as in Table II.

In conclusion, the real time autofluorescence endoscopy is a promising system to diagnose dysplastic lesions and early carcinomas in the GI tract as an adjunct to ordinary white light endoscopy. Moreover, this system, which needs no administration of a photosensitive agent, may be of use in screening for the early detection of cancers and/or pre-cancerous lesions.

The prototype LIFE-GI system, however, has several problems to be solved in order to apply this system to close examination of lesions. Instrumental false positive and negative imagings occurring mainly due to limited sensitivity of photodetectors, background quenching and photobleaching (loss of fluorescence intensity after light exposure) are among the problems to be solved. It is also important for the improvement of the system to identify the fluorescent molecules and clarify spectral properties of the molecules in determining pathological lesions.

### *Acknowledgments*

This study was supported in part by a Grant-in-Aid for Cancer Research (No. 10-37) from the Ministry of Health and Welfare of Japan.

### *References*

[1] Palcic, B., Lam, S., Jung, J. and MacAulay, C. Detection and localization of early lung cancer by imaging techniques. *Chest* 1991; **99**: 742–743.

[2] Schomacker, K.T., Frisoli, J.K., Compton, C.C. *et al.* Ultraviolet laser-induced fluorescence of colonic polyps. *Gastroenterology* 1992; **102**: 1155–1161.

[3] Kapadia, C.R., Cutruzzola, F.W., O'Brien, K.M. *et al.* Laser-induced fluorescence spectroscopy of human colonic mucosa: detection of adenomatous transformation. *Gastroenterology* 1990; **99**: 150–157.

[4] Cohren, R.M., Richards-Kortum, R., Sivak, M.V. *et al.* Gastrointestinal tissue diagnosis by laser-induced fluorescence spectroscopy at endoscopy. *Gastrointest. Endosc.* 1990; **36**: 105–111.

[5] Panjehpour, M., Overholt, B.F., Schmidhammer, J.L. *et al.* Spectroscopic diagnosis of esophageal cancer: new classification model, improved measurement system. *Gastrointest. Endosc.* 1995; **41**: 577–581.

[6] Panjehpour, M., Overholt, B.F., Vo-Dinh, T. *et al.* Endoscopic fluorescence detection of high-grade dysplasia in Barret's esophagus. *Gastroenterology* 1996; **111**: 93–101.

[7] Wang, T.D., Dam, J.V., Crawford, J.M. *et al.* Fluorescence endoscopic imaging of human colonic adenomas. *Gastroenterology* 1996; **111**: 1182–1191.

[8] Zonios, G.I., Cothren, R.M., Arendt, J.T. *et al.* Morphological model of human colon tissue fluorescence. *IEEE Transactions on Biomedical Engineering* 1996; **43**: 113–122.

[9] Chwirot, B.W., Chwirot, S., Jedrzejczyk, W. *et al.* Ultraviolet laser-induced fluorescence of human stomach tissues: detection of cancer tissues by imaging techniques. *Lasers Surg. Med.* 1997; **21**: 149–158.

[10] Vo-Dinh, J., Panjehpour, M., Qverholt, B.F. *et al.* Laser-induced differential fluorescence for cancer diagnosis without biopsy. *Applied Spectroscopy* 1997; **51**: 58–63.

[11] Yano, H., Iishi, H. and Tatsuta, M. Diagnosis of early gastric cancers by an endoscopic autofluorescence imaging system. *Endoscopy* 1996; **28**: S29.

[12] DuVall, G.A., Kost, J., Scheider, D. *et al.* Laser induced fluorescence (LIF) endoscopy (E): a pilot study of a real-time (RT) autofluorescence imaging system for early detection of dysplasia and carcinoma in the gastrointestinal (GI) tract. *Endoscopy* 1996; **28**: S45.

[13] van Ierland, M.L. and Tytgat, G.N.J. Detection of dysplasia using fluorescence *in vivo* using the Xillix-Life-GI system. *Endoscopy* 1996; **28**: S44–S45.

[14] Watanabe, H., Ogihara, T., Namihisa, A. *et al.* Application of a new fluorescence endoscopic imaging system to the detection of small remnant of endoscopically resected gastric neoplasm. *7th Asian-Pacific Congress of Digestive Endoscopy* 1996; 124.

[15] Zeng, H., Weiss, A., MacAulay, C.E. *et al.* Development of a fluorescence video endoscopy imaging system for the early detection of cancer in the gastrointestinal tract. *SPIE* 1997; **2976**: 291–296.

[16] Namihisa, A., Watanabe, H., Ogihara, T. *et al.* Comparison of fluorescent spectra between normal mucosa and gastric adenocarcinomas under a real timed autofluorescence endoscopy. *Gastroint. Endosc.* 1998; **47**: AB36.

[17] Watanabe, H., Ogihara, T., Namihisa, A. *et al.* Usefulness of a new autofluorescence endoscopic imaging system for the diagnosis of gastric neoplasm. *Endoscopy* 1996; **28**: S68.

[18] Watanabe, H., Ogihara, T., Namihisa, A. *et al.* Real-time diagnosis of superficial type early colo-rectal cancers using an auto-fluorescence endoscopic system. *Gastroint. Endosc.* 1998; **47**: AB41.

